# Comparison of Augmented Reality‐Based and Conventional Training Methods for Radiographic Positioning in Second‐Year Radiologic Technology Students in Japan

**DOI:** 10.1002/jmrs.70019

**Published:** 2025-08-22

**Authors:** Shin Nagamata

**Affiliations:** ^1^ Department of Radiological Technology, Faculty of Fukuoka Medical Technology Teikyo University Omuta Fukuoka Japan; ^2^ Department of Radiological Technology, Faculty of Health Science Kobe Tokiwa University Kobe Hyogo Japan

**Keywords:** augmented reality, patient positioning, radiographer, radiography, random allocation

## Abstract

**Introduction:**

In Japanese training schools for radiologic technologists, improving students' practical skills presents an educational challenge. To address this issue, an augmented reality (AR)‐based visual assistance tool for radiographic positioning training was developed. This study aimed to evaluate and compare the effectiveness of a previously developed AR tool with conventional training methods.

**Methods:**

Fifty‐seven second‐year radiologic technology students were randomly divided into two groups: an experimental group (*n* = 29) trained with AR and a control group (*n* = 28) trained with physical auxiliary tools. Both groups practised positioning the torso at 35° and 45° for 1.5 min each. The timeframe aligned with the typical time allocated to each student during traditional hands‐on training. Proficiency tests were conducted 1 and 4 weeks after training to evaluate the accuracy of the angles.

**Results:**

In the test conducted 1 week after training, the experimental group demonstrated significantly smaller errors than the control group (*p* = 0.002). However, in the test administered 4 weeks after training, although the experimental group continued to show smaller errors, the difference was not statistically significant (*p* = 0.066).

**Conclusion:**

AR‐based training can enhance radiographic positioning training. However, a single brief session is considered insufficient for maintaining long‐term learning effects. Continuous use of AR is warranted for achieving long‐term educational effectiveness. Future research is needed to optimise AR training methods and determine the impact on students' clinical training performance and postgraduate proficiency.

## Graphical abstract

This study evaluated the effectiveness of augmented reality (AR)‐based radiographic positioning training compared to conventional training using physical auxiliary tools. The AR group (*n* = 29) demonstrated a significantly greater reduction in positioning angle errors than the control group (*n* = 28) at 1 week after training (*p* = 0.002); however, the improvement was comparable between the groups 4 weeks later (*p* = 0.066).

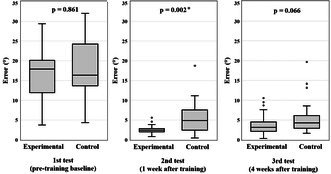

## 1 Introduction

Radiographers have to demonstrate high levels of accuracy while positioning patients, as a slight over‐rotation or misalignment of an affected area can result in poor image quality and complicate the interpretation of the radiographic image [1]. Moreover, radiologic technologists are required to comply with radiation safety protocols, ensuring that the image is acquired correctly on the first attempt to avoid unnecessary radiation exposure [2]. Standard positions help radiologists interpret radiographic images in relation to anatomical structures [3]. Thus, radiographic positioning is a challenging task that students experience in clinical practice, as it is directly related to the acquisition of high‐quality radiographic images and the control of patient radiation exposure.

In the Japanese radiologic technology curriculum, the number of credits allocated to clinical practice has been increased for students joining from the academic year 2022. In addition, the style of clinical training has begun to change from observation to participation‐based, and improving students' practical skills has become an educational issue. The reasons for curriculum modification include the ageing of the population, increasing and diversifying healthcare needs, and the evolving role of radiologic technologists due to the promotion of team‐based healthcare [4].

In Europe, where participatory clinical practice is already well‐established, simulation training in virtual environments has been widely adopted [5]. Virtual simulation offers the advantage of allowing students to train safely without the radiation risk and the time constraints involved in using physical radiographic laboratories. Rowe et al. [6] have classified virtual reality (VR) experiences into non‐immersive, semi‐immersive and fully immersive types based on the level of user immersion. Non‐immersive VR is screen‐based and operated using keyboards and mice, resulting in minimal immersion. Semi‐immersive VR provides a moderate level of immersion, typically achieved through large projectors or multiple monitors. Fully immersive VR uses a head‐mounted display and hand controllers to fully engage users in a virtual environment.

In recent years, research on the efficacy of fully immersive VR simulations has increased in radiography education. O'Connor and Rainford [7] investigated the translation of VR learning into student performance by evaluating clinical assessment grades in either upper or lower extremity radiography and showed that the VR group performed better than the no VR education group, particularly in patient positioning, exposure parameter selection and image appraisal skills. Rowe et al. [6] compared the Objective Structured Clinical Examination (OSCE) assessment outcomes of students trained with VR and physical simulations. The results indicated that the VR group made fewer errors in equipment setup and positioning and showed a significant reduction in the OSCE time. Sapkaroski et al. [8] compared the hand‐positioning performance of students trained with VR simulation and those trained with conventional clinical role‐play in a radiography laboratory. The results of the study showed that the VR group scored significantly better in digit separation, palm flatness and central ray position. However, the realism of palpation and patient interaction is limited because the VR patient is controlled by hand controllers in the VR environment. Kato et al. [9] investigated the effect of VR constraints on radiographic skill proficiency in a real‐world setting using posterior–anterior chest and lateral elbow radiography. In this study, although the VR training group had a poorer positioning performance than the physical training group, the difference was not statistically significant. However, the VR group performed significantly worse in terms of the placement of the X‐ray beam, image detector and side marker. In addition, the items ‘hospitality and concern for patient pain’ and ‘process control and safety’ were rated significantly lower. Nevertheless, the validity of the results reported by Kato et al. is debatable. Gardling et al. [10] suggested that the poorer performance of the VR group in Kato et al.'s study could be attributed to students training independently without feedback and receiving only basic instruction on the functioning of the system. Thus, the published literature presents mixed views on the usefulness of VR simulations in radiography.

Limited research has addressed one of the key shortcomings of fully immersive VR simulation, which requires users to manipulate a virtual patient using hand controllers. Unlike VR, augmented reality (AR) overlays virtual objects onto the real world. The user's perception comes from the real environment rather than the virtual environment [11, 12]. Raith et al. [3] proposed a training method for radiographic positioning using AR to overcome the limitation of VR. In the training environment, a hologram of a correctly positioned body part is projected onto the real world, and the trainee uses it as a reference while positioning. The system was designed to enable positioning training that was less dependent on access to laboratories and instructors. The quantitative effectiveness of the system was not evaluated. However, in their study, user tests with radiography instructors using projections of the scaphoid bone and both hands demonstrated that AR‐assisted training is a useful addition to conventional educational methods.

However, the aforementioned literature did not analyse radiographic positioning for an oblique view. The oblique view has the characteristic of rotating the affected area at a certain angle relative to the imaging detector. To learn the positioning angle, it is essential to practise setting the angle repeatedly with both hands and memorising the space between the examination area and image detector, as seen from their individual heights. Therefore, it is challenging to provide sufficient support solely by operating a VR patient with hand controllers or presenting a hologram using AR. In traditional radiographic laboratory training, students often have limited time to practise setting the positioning angles because of the need to perform multiple tasks, including positioning for various views, patient interaction, equipment operation, exposure parameter selection and image evaluation. Consequently, they are typically taught using auxiliary tools, such as positioning blocks and angle gauges, which can hinder hands‐on practice by occupying one hand.

To address this problem, an AR‐based radiographic positioning training tool was developed in a previous study [13]. This tool displays a virtual angle gauge on the camera feed by detecting a fiducial marker using a webcam connected to a laptop. Using the virtual angle gauge as a visual aid, trainees can practice oblique positioning without relying on auxiliary physical tools. The previous study indicated that AR technology could be applied as an instructional aid in radiographic laboratory training. However, the educational effectiveness of the AR‐based training had not yet been quantitatively evaluated. If the system proposed by Raith et al. is classified as immersive AR, as it overlays holograms directly onto the real world, our system can be categorised as non‐immersive AR because it presents three‐dimensional (3D) computer graphics (CG) on a monitor. To the best of my knowledge, this is the first study to address instructional support focused on setting positioning angles using a combination of physical lab training and AR.

The present study aimed to conduct a follow‐up evaluation of the previous study by quantitatively assessing the learning effectiveness of AR‐based training. The goal of this work is to contribute to improving students' practical skills by providing a different approach that complements traditional hands‐on radiography training, distinct from existing VR and AR training methods.

## 2 Methods

This study was an intervention study that employed randomisation. As the author independently conducted all phases of the research process, blinding for the intervention delivery, outcome assessment and data analysis were not feasible.

**FIGURE 1:**
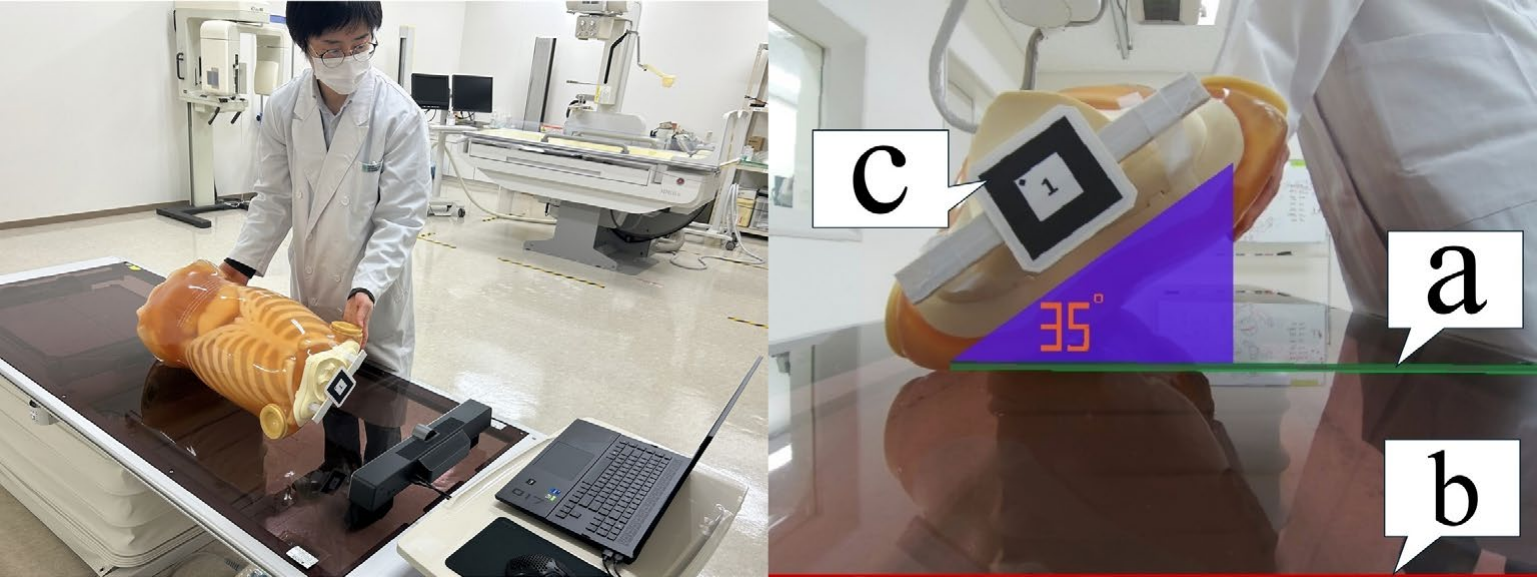
AR‐based training method. The anthropomorphic phantom is positioned by aligning the line extending from the base of the triangular 3D‐CG object (a) with a reference line at the bottom of the camera feed (b). Label (c) indicates a fiducial marker used to display the virtual object. The virtual angle gauge can also be relocated anywhere on the feed by clicking; thereby enhancing flexibility during training.

The evaluation targets for this study were torso positioning angles of 35° and 45°, which were the same as those employed in the previous study. These angles were selected because they are commonly used in radiographic procedures requiring oblique projections, such as lumbar spine obliques (for zygapophyseal joints), Lauenstein views of the hip, and Judet views of the acetabulum. These views require precise rotation of the patient to avoid anatomical superimposition and are known to be technically challenging for students. Owing to the greater weight of the torso compared to that of other anatomical areas, positioning with traditional tools such as a positioning block or angle gauge presents certain challenges. A positioning block occupies one hand, limiting free movement, whereas an angle gauge requires the trainee to lean repeatedly to check the scale for each adjustment, making the process time‐consuming and less efficient. By contrast, the virtual angle gauge visually guides the trainee through AR technology, allowing them to practise with both hands free and eliminating the need to tilt their body or lean in to check the scale. Figure 1 shows an example of AR‐based training. In the previous study, the positioning angle was set by making the line extending from the base of the triangular 3D‐CG object (labelled a in Figure 1) appear horizontal in the camera feed. In the present study, a reference line (labelled b in Figure 1) was added to the bottom of the camera feed. The trainee sets the angle by aligning the line extending from the base of the triangle (a) with this reference line (b). c in Figure 1 indicates the fiducial marker used to display the virtual angle gauge. The virtual angle gauge was created using the 3D modelling software Metasequoia 4 [14], and the AR environment was developed using the ARToolkit [15], an open‐source C++ library for AR applications. The hardware used for this study included a laptop running Windows 11 Pro 64‐bit, equipped with an Intel Core i7‐12800HX CPU, NVIDIA GeForce RTX 3070 Ti Laptop GPU, a 17.3‐in. display (3840 × 2160 resolution), and 16GB of graphics memory. The webcam used for fiducial marker detection was MAXHUB Soundbar SE.

### 2.1 Participants

This study was approved by the Ethics Committee of the Faculty of Fukuoka Medical Technology at Teikyo University (Approval No. Teifukurin 23‐06). Written informed consent was obtained from all participants, and data anonymity was ensured. The participants were second‐year students from the Department of Radiological Technology in the Faculty of Fukuoka Medical Technology at Teikyo University. At this university, students receive theoretical instruction in radiography during the second semester of their first year of study. Laboratory instruction begins in the first semester of the second year, covering chest, abdominal and head imaging, whereas spine, pelvis, hip joint and extremity imaging are taught in the second semester. Clinical placements are undertaken in the second semester of their third year. Given that this study was conducted during the first semester, participants had theoretical knowledge of radiography but no hands‐on experience with oblique positioning of the torso. In addition, a distinctive feature of the Japanese radiologic technology curriculum is that students receive cross‐disciplinary education in diagnostic imaging, radiation therapy and nuclear medicine, as radiologic technologists in Japan are responsible for all three areas. The study participants had begun learning diagnostic imaging in their second year, with radiation therapy and nuclear medicine scheduled for their third year.

Convenience sampling was used in this study. Students from the academic years 2023 and 2024 were recruited via public announcements, resulting in a total of 57 participants. The admission requirements and degree programmes were identical for both academic years. Participants were randomly assigned to two groups using computer‐generated random numbers. Simple randomisation was applied to participants from the academic year 2023. Block randomisation was implemented for the academic year 2024 to ensure balanced group sizes. Of the 57 participants, 29 students were allocated to the experimental group, which was trained using the virtual angle gauge, while 28 students were assigned to the control group, which was trained using physical auxiliary tools. The experimental group included 10 students (5 men and 5 women) from the 2023 cohort and 19 students (14 men and 5 women) from the 2024 cohort. The control group comprised 5 students (3 men and 2 women) from the 2023 cohort and 23 students (17 men and 6 women) from the 2024 cohort. The characteristics of the participants are summarised in Table 1. Participants' grades in the radiographic theory course were examined as potential confounding factors. This course was graded on a five‐point scale ranging from S (excellent) to D (failure). For analytical purposes, S was assigned a value of 4 and D a value of 0.

**TABLE 1:** Characteristics of the study population.

Variable	Experimental group (*n* = 29)	Control group (*n* = 28)
Male (2023 cohort, 2024 cohort)	19 (5, 14)	20 (3, 17)
Female (2023 cohort, 2024 cohort)	10 (5, 5)	8 (2, 6)

### 2.2 Training Methods

The training methods used for each group are described below. The use of each tool was explained immediately before the training, and no instructor intervention was provided during the training.

#### 2.2.1 Experimental Group

1. The angle was positioned multiple times using the virtual angle gauge as a reference.
2. After developing the sensation of the angle, the positioning angle was set without referring to a virtual angle gauge.
3. The trainee paused at the angle set in step 2 and checked for any excess or deficiency by referring to the virtual angle gauge.
4. Steps 1–3 were repeated to identify and correct any positioning tendencies. Students independently repeated these steps at their own pace.

#### 2.2.2 Control Group

1. The angle was positioned multiple times using a physical angle gauge and positioning block.
2. After determining the angle, the positioning angle was set without using auxiliary tools.
3. The trainee paused at the angle set in step 2 and checked for any excess or deficiency by using auxiliary tools.
4. Steps 1–3 were repeated to identify and correct any positioning tendencies. Students independently performed these steps at their own pace.

### 2.3 Experimental Procedure

**FIGURE 2:**
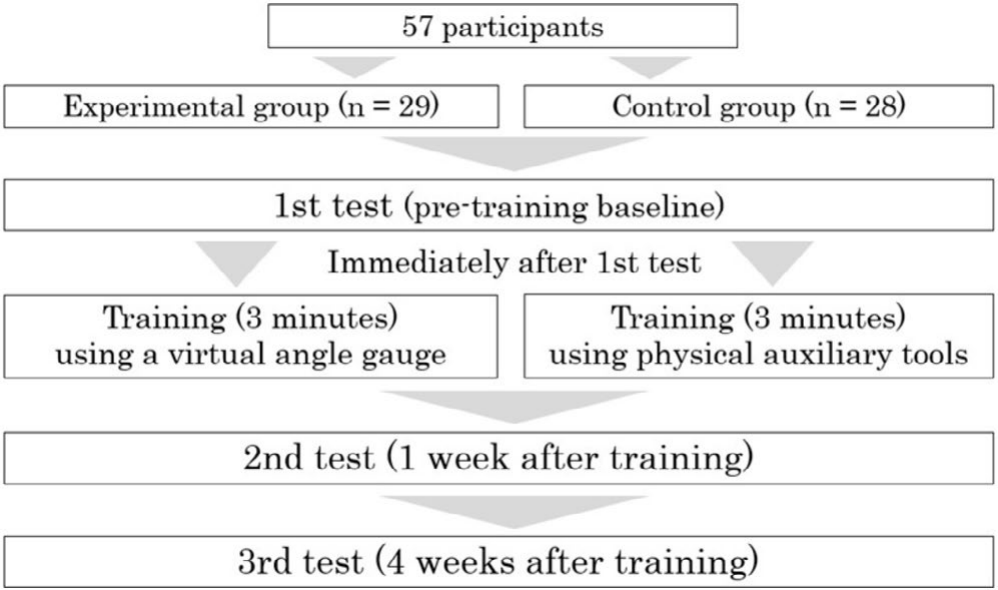
Experimental procedure.

The details of the experimental procedure are outlined below (Figure 2). Participants were allowed to engage in additional practice using both training tools following the completion of all experimental procedures.

1. First test (pre‐training baseline): Before training, the baseline positioning accuracy was measured. The participants were instructed to rotate the anthropomorphic phantom to what they considered as 35° and 45°, stopping their hands at the respective positions. The angles were captured using a webcam and measured using ImageJ, an image analysis software developed by the National Institutes of Health.
2. Training lmmediately: following the first test, the participants practised positioning at 35° and 45° for 1.5 min each for a total of 3 min. This timeframe aligned with the typical time allocated to each student in traditional practical training sessions.
3. Second test (1 week after training): A proficiency test identical to the first test was conducted 1 week after training to evaluate retention.
4. Third test (4 weeks after training): The same test was administered 4 weeks after training to assess the retention of positioning accuracy.

### 2.4 Statistical Analysis

Positioning accuracy was evaluated using the following formula:

$$\text{Error}=\frac{\left(\left|35^{{}^{\circ}}-\text{angle}\;\mathrm{set}\;\mathrm{by}\;\text{participant}\right|+\left|45^{{}^{\circ}}-\text{angle}\;\mathrm{set}\;\mathrm{by}\;\text{participant}\right|\right)}{2}$$

Smaller errors indicate higher accuracy and better performance. As the Shapiro–Wilk test indicated that the data were non‐normally distributed (*p* < 0.05), differences in errors between the groups across the three tests were compared using the Mann–Whitney *U* test. Effect sizes (ES) and 95% confidence intervals (CI) were calculated for the second and third tests. Similarly, differences in the participants' radiographic theory grades were analysed using the *U* test. The Shapiro–Wilk and Mann–Whitney *U* tests were performed using IBM SPSS 28 Advanced Statistics (IBM Corp., Armonk, NY), and ES and CI were calculated using Python 3.11.5 (Python Software Foundation, Wilmington, DE).

## 3 Results

**FIGURE 3:**
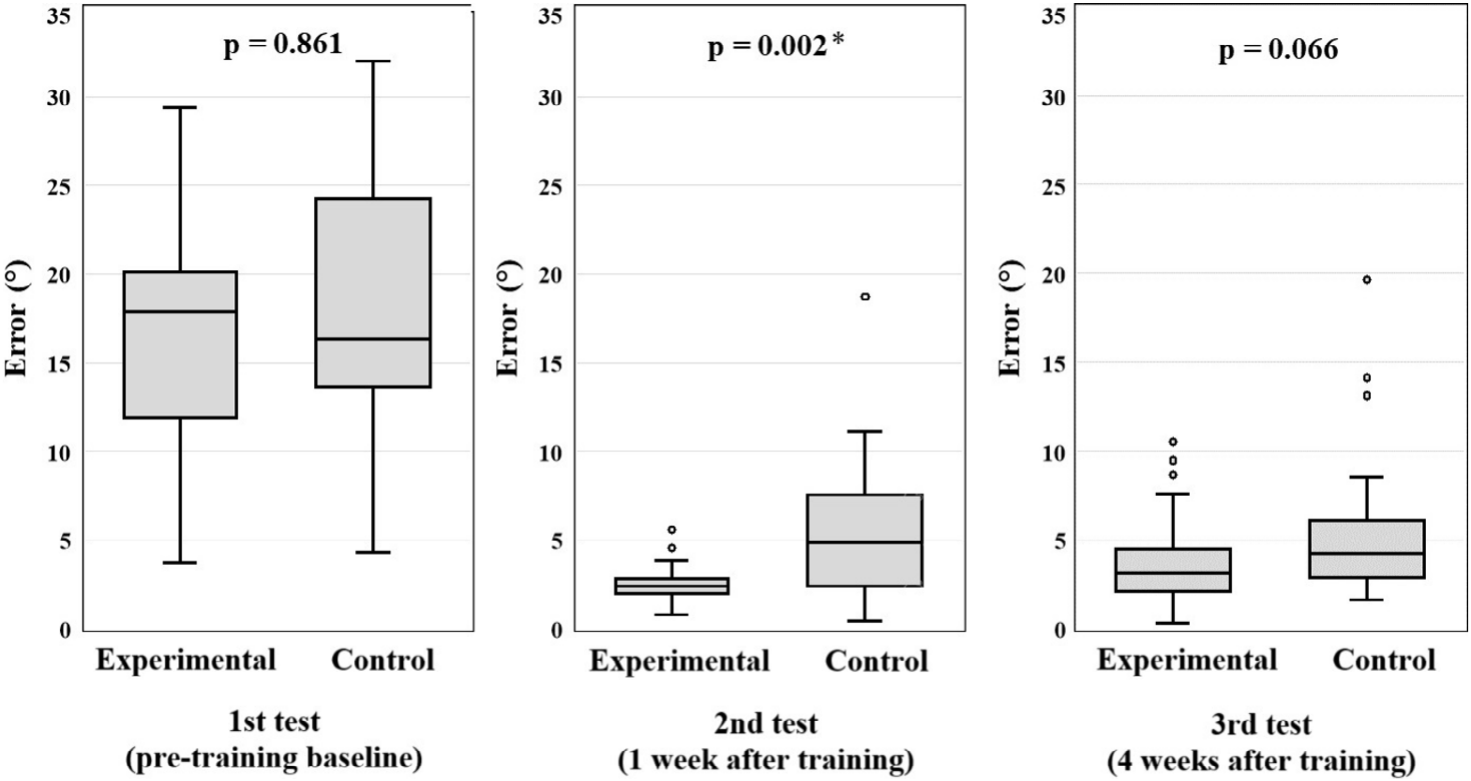
Transition of the angle errors across the three tests.

All 57 participants completed the study, and no dropouts occurred during the study period. Table 2 presents the radiographic theory course grades. There was no significant difference in grades between the two groups (*U* = 402, *z* = −0.71, *p* = 0.943). Table 3 and Figure 3 show the results of the three tests for each group. In the 1st test, the experimental group showed a larger positioning error than the control group; however, this difference was not statistically significant (*U* = 395, *z* = −0.176, *p* = 0.861). By the second test, the experimental group demonstrated significantly smaller errors than the control group (*U* = 214, *z* = −3.065, *p* = 0.002) (*r* = 0.41, 95% CI: 0.16–0.60). In the third test, although the experimental group continued to exhibit smaller errors, the difference was not statistically significant (*U* = 291, *z* = −1.836, *p* = 0.066) (*r* = 0.24, 95% CI: −0.02 to 0.47).

**TABLE 2:** Radiographic theory course grades.

Group	Radiographic theory course grade (*μ* ± *σ*)
Experimental	1.59 ± 1.09
Control	1.50 ± 0.69

*Note:* This course used a five‐point grading scale ranging from S (excellent) to D (failure). For analysis, S was assigned a value of 4 and D a value of 0.

**TABLE 3:** Descriptive statistics for positioning errors of the groups using the three tests.

	Median		Mean ± SD	
	Experimental	Control	Experimental	Control
First test	17.93	16.37	16.7 ± 6.46	17.42 ± 7.39
Second test	2.44	4.86	2.66 ± 1.11	5.62 ± 3.99
Third test	3.12	4.27	3.73 ± 2.55	5.39 ± 4.16

## 4 Discussion

Japan is a super‐ageing society facing severe labour shortages. The Ministry of Health, Labour, and Welfare estimates that by 2025, one in three people will be aged ≥ 65 years and one in five will be aged ≥ 75 years [16]. Developing professionals equipped with advanced practical skills is an urgent priority for radiologic technologist training schools. To address this challenge, this study proposed an AR‐based radiographic positioning training method and evaluated its effectiveness in improving positioning skills.

These findings revealed that the experimental group showed a significant reduction in positioning angle errors compared to the control group in the second test conducted 1 week after the training. Although the ES was classified as medium [17], the observed improvement is educationally meaningful as even slight rotational misalignment of the affected area can lead to anatomical superimposition, potentially compromising the diagnostic accuracy of radiographic images. This improvement can be attributed to the visual assistance provided by AR, which allowed students to use both hands freely, thereby enhancing practice efficiency. Furthermore, the ability to easily identify the angular misalignment on a monitor facilitates an effective trial‐and‐error process. The training procedure (steps 1–4) implemented in this study reflects Kolb's experiential learning theory [18]. This theory posits that learning is enhanced through four cyclical stages: concrete experience, reflective observation, abstract conceptualisation and active experimentation. The author considers that AR contributed to the effective functioning of this cycle. However, 4 weeks after the training, the effect of AR was comparable to that of the conventional method, which may be attributed to the fact that the training was conducted only once and lasted for just 3 min. In a systematic review of immersive VR training in radiography education, Gardling et al. [10] listed the length of the VR session as one of the key factors contributing to successful outcomes. Arroyo and Garcia [19] integrated immersive VR training into the curriculum of a 3‐year degree programme to investigate its long‐term effectiveness. Their results demonstrated that hybrid training, which incorporates VR and physical simulation, achieved superior educational outcomes over traditional physical simulation alone. VR training enables students to repeatedly experience successful and failed radiographic procedures, aligning with Kolb's four stages of experiential learning [19, 20]. Based on these findings, AR, similar to VR, may yield greater long‐term learning effects than training with physical auxiliary tools, provided it is used continuously. The training timeframe in this study was designed based on the typical time allocated to each student in laboratory sessions. Even with just 3 min of practice, using AR in sessions targeting different anatomical areas, such as the torso, shoulder and cervical spine, could help students develop a better sense of positioning angles. In the present study, AR was shown to be as effective as the conventional training methods at 4 weeks post‐training. However, the sustained improvement in skills observed in both groups is encouraging from an educational perspective.

In recent years, several studies have highlighted the educational value of VR and recommended its integration into radiography education curricula [6, 7, 19, 21, 22]. The author concurs with this perspective and further emphasises the distinctive educational potential of AR. Immersive VR simulation requires a dedicated space and is conducted as a standalone training method, separate from physical simulation. In contrast, AR can be integrated into physical simulation settings and used as an instructional aid, allowing its flexible incorporation into traditional educational frameworks. Furthermore, several free AR applications that can display user‐created 3D models help reduce implementation costs. In this study, Metasequoia was used to create virtual angle gauges, and ARToolKit was used for fiducial marker detection and 3D model display; both tools are freely available.

Additionally, AR enables trainees to perform tasks in the real world rather than in a virtual environment, thereby supporting training through an approach that differs from VR. AR has the potential to serve as an alternative to VR, particularly for training that requires trainees to use visual and tactile perception, such as oblique radiographic positioning. However, AR may increase cognitive load due to the simultaneous processing of real and virtual elements. Prior research has shown that AR can be more distracting than immersive VR in a learning context that requires visual and auditory attention [23]. This cognitive load issue should be carefully considered when designing AR‐based radiography education. For example, in positioning training, focusing on technical skills may be more appropriate than incorporating comprehensive tasks that involve communication skills.

Another approach to improve students' practical skills is the use of non‐immersive VR simulations in lectures. Training in non‐patient interaction skills, such as equipment setup and image evaluation in non‐immersive VR environments, is as effective as [20] or even more effective [24, 25, 26] than traditional educational approaches. User feedback was highly positive [20, 27, 28]. In particular, VR training, which enables students to safely and repeatedly correct positioning errors, provides substantial educational value for the interpretation of radiographic images. As described by Miller et al. [20], VR enhances access to authentic practice and offers unique learning opportunities that are not available through traditional training methods. Insights from countries with well‐established participatory clinical practice should provide valuable guidance for addressing educational challenges in Japan.

To the best of my knowledge, this is the first study to quantitatively evaluate the effectiveness of AR in radiography education. However, this study only evaluated the accuracy of the positioning angles. In clinical practice, radiographers must also consider joint twisting and the appropriate application of force while positioning patients. Therefore, during the experiment, the scope of training using the training tools was clearly explained to the students, and careful guidance was provided. Efforts are underway to develop training tools that offer comprehensive instructions regarding radiographic positioning skills. Future research is needed to determine the impact of this study on students' clinical training performance and postgraduate proficiency.

In this study, AR‐based visual assistance was employed to train radiographic positioning, a skill that improves with repeated practice. In addition, students highly valued this instructional method [13] because of its ease of training and usefulness. Therefore, AR‐based visual assistance was considered to have potential applications across various educational fields, including radiographic positioning.

## 5 Conclusion

The AR group demonstrated significantly smaller positioning errors than the physical auxiliary tool group 1 week after training. However, the improvement was comparable between the two groups 4 weeks later. Continuous use of AR, rather than a single brief session, is necessary for achieving long‐term educational effectiveness.

AR‐based radiographic positioning training has the potential to enhance learning outcomes. Further research is required to optimise AR training methods and to determine the impact on students' clinical training performance and their postgraduate proficiency.

## Ethics Statement

Ethics approval was granted by the Ethics Committee of the Faculty of Fukuoka Medical Technology, Teikyo University (approval no. Teifukurin 23‐06).

## Consent

Written informed consent was obtained from all participants, and data anonymity was ensured.

## Conflicts of Interest

The author declares no conflicts of interest.

## Data Availability

Anonymised data that support the findings of this study are available from the corresponding author upon reasonable request.
